# Heterogeneities in Ventricular Conduction Following Treatment with Heptanol: A Multi-Electrode Array Study in Langendorff-Perfused Mouse Hearts

**DOI:** 10.3390/life12070996

**Published:** 2022-07-05

**Authors:** Xiuming Dong, Gary Tse, Guoliang Hao, Yimei Du

**Affiliations:** 1 Henan SCOPE Research Institute of Electrophysiology Co., Ltd., Kaifeng 475000, China; xiuming.dong@epscopelab.com (X.D.); guoliang.hao@epscopelab.com (G.H.); 2 Cardiac Electrophysiology Unit, Cardiovascular Analytics Group, Hong Kong, China; gary.tse@kmms.ac.uk; 3 Tianjin Key Laboratory of Ionic-Molecular Function of Cardiovascular Disease, Department of Cardiology, Tianjin Institute of Cardiology, Second Hospital of Tianjin Medical University, Tianjin 300211, China; 4 Kent and Medway Medical School, Canterbury CT2 7FS, UK; 5 Burdon Sanderson Cardiac Science Centre, BHF Centre of Research Excellence, Department of Physiology, Anatomy and Genetics, University of Oxford, Oxford OX1 3PT, UK; 6 Department of Cardiology, Union Hospital, Tongji Medical College, Huazhong University of Science and Technology, Wuhan 430022, China; 7 Research Center of Ion Channelopathy, Institute of Cardiology, Union Hospital, Tongji Medical College, Huazhong University of Science and Technology, Wuhan 430022, China

**Keywords:** action potential duration, variability, entropy, detrended fluctuation analysis, hypokalemia, conduction, heterogeneity, inhomogeneity, dispersion, mouse, heptanol

## Abstract

Background: Previous studies have associated slowed ventricular conduction with the arrhythmogenesis mediated by the gap junction and sodium channel inhibitor heptanol in mouse hearts. However, they did not study the propagation patterns that might contribute to the arrhythmic substrate. This study used a multi-electrode array mapping technique to further investigate different conduction abnormalities in Langendorff-perfused mouse hearts exposed to 0.1 or 2 mM heptanol. Methods: Recordings were made from the left ventricular epicardium using multi-electrode arrays in spontaneously beating hearts during right ventricular 8 Hz pacing or S1S2 pacing. Results: In spontaneously beating hearts, heptanol at 0.1 and 2 mM significantly reduced the heart rate from 314 ± 25 to 189 ± 24 and 157 ± 7 bpm, respectively (ANOVA, *p* < 0.05 and *p* < 0.001). During regular 8 Hz pacing, the mean LATs were increased by 0.1 and 2 mM heptanol from 7.1 ± 2.2 ms to 19.9 ± 5.0 ms (*p* < 0.05) and 18.4 ± 5.7 ms (*p* < 0.05). The standard deviation of the mean LATs was increased from 2.5 ± 0.8 ms to 10.3 ± 4.0 ms and 8.0 ± 2.5 ms (*p* < 0.05), and the median of phase differences was increased from 1.7 ± 1.1 ms to 13.9 ± 7.8 ms and 12.1 ± 5.0 ms by 0.1 and 2 mM heptanol (*p* < 0.05). P_5_ took a value of 0.2 ± 0.1 ms and was not significantly altered by heptanol at 0.1 or 2 mM (1.1 ± 0.9 ms and 0.9 ± 0.5 ms, *p* > 0.05). P_50_ was increased from 7.3 ± 2.7 ms to 24.0 ± 12.0 ms by 0.1 mM heptanol and then to 22.5 ± 7.5 ms by 2 mM heptanol (*p* < 0.05). P_95_ was increased from 1.7 ± 1.1 ms to 13.9 ± 7.8 ms by 0.1 mM heptanol and to 12.1 ± 5.0 ms by 2 mM heptanol (*p* < 0.05). These changes led to increases in the absolute inhomogeneity in conduction (P_5–95_) from 7.1 ± 2.6 ms to 31.4 ± 11.3 ms, 2 mM: 21.6 ± 7.2 ms, respectively (*p* < 0.05). The inhomogeneity index (P_5–95_/P_50_) was significantly reduced from 3.7 ± 1.2 to 3.1 ± 0.8 by 0.1 mM and then to 3.3 ± 0.9 by 2 mM heptanol (*p* < 0.05). Conclusion: Increased activation latencies, reduced CVs, and the increased inhomogeneity index of conduction were associated with both spontaneous and induced ventricular arrhythmias.

## 1. Introduction

Conduction velocity (CV) is an important variable for the propagation of cardiac action potentials (APs) [1,2]. Defects in conduction are found with aging [3], and in many pathological states, such as heart failure [4], diabetic cardiomyopathy [5], long QT syndrome [6], and Brugada syndrome [7,8]. Therefore, the elucidation of the relative contributions of abnormal conduction to the arrhythmic substrate can provide opportunities for the development of novel pharmacotherapy that can potentially restore conduction. Gap junctions and sodium channels are the main ion channels that govern the cardiac CV [9,10,11,12]. Heptanol is a drug that uncouples gap junctions at concentrations < 2 mM and additionally inhibits sodium channels > 2 mM [13]. It has previously been used to explore the contributions of conduction abnormalities to ventricular arrhythmogenesis in different animal models [14,15]. In our previous work, we associated the arrhythmogenic effects of heptanol to reduced CVs [16], abnormalities in action potential duration (APD) and CV restitution [17], as well as alterations in beat-to-beat repolarization variability in repolarization, using monophasic action potential (MAP) recordings [18,19]. However, CV was reduced by similar extents in arrhythmic and non-arrhythmic hearts, which would suggest factors other than reduced CVs were predisposing to arrhythmogenesis. We hypothesized that increased conduction heterogeneities may be a contributory factor, but the use of MAP recordings does not allow for the visualization or the measurement of local activation and propagation through the myocardium or recording from multiple sites simultaneously [20]. By contrast, the multielectrode array allows the simultaneous recording of electrical activity from multiple sites and the reconstruction of activation maps from the recorded signals [21]. Therefore, this study used a multi-electrode array mapping technique to further investigate the following abnormalities of reduced CV: the increased temporal and spatial dispersion of conduction as determined by the standard deviation of local activation times and inhomogeneity indices in Langendorff-perfused mouse hearts exposed to 0.1 or 2 mM heptanol. Using this platform, it is possible to investigate the effects of exogenous substances free from endogenous modulators released by the nervous system [22,23,24,25]. Thus, the experimental design allowed us to test the hypothesis that conduction abnormalities contribute to the arrhythmic substrate.

## 2. Materials and Methods

### 2.1. Solutions

Krebs–Henseleit solution (composition in mM: NaCl 119, NaHCO_3_ 25, KCl 4, KH_2_PO_4_ 1.2, MgCl_2_ 1, CaCl_2_ 1.8, glucose 10, and sodium pyruvate 2, pH 7.4), which had been bicarbonate-buffered and bubbled with 95% O_2_–5% CO_2_, was used in the experiments described in this study. Heptanol (Sigma, Dorset, UK; density: 0.82 g mL^−1^) is an agent that remains soluble in aqueous solutions up to 9 mM (The Merck Index, Hoboken, NJ, USA). Krebs–Henseleit solution was used to dilute the heptanol solution to produce a final concentration of 0.1 mM.

### 2.2. Preparation of Langendorff-Perfused Mouse Hearts

All experiments involving animals were approved by the Animal Research Ethics Committee of Tongji Medical College, Huazhong University of Science and Technology (IACUC Number: 2307) and were carried out in accordance with the National Institutes of Health Guide for the Care and Use of Laboratory Animals (NIH Publication, revised 2011). Male C57BL/6 mice were purchased from Vital River Laboratories, Beijing, China. Mice between 5 and 7 months of age were used (*n* = 5). They were maintained at room temperature (21 ± 1 °C) and were subjected to a 12:12 h light/dark cycle with free access to sterile rodent chow and water in an animal facility. Mice were anesthetized with isoflurane. The hearts were removed from their chest cavities and then submerged in ice-cold Krebs–Henseleit solution. The aortas were cannulated using a custom-made 21-gauge cannula prefilled with ice-cold buffer. A micro-aneurysm clip was used to secure the hearts onto the Langendorff perfusion system. Retrograde perfusion was carried out at a flow rate of 2 to 2.5 mL min^−1^ by use of a peristaltic pump. The perfusate passed successively through 200 and 5 μm filters and was warmed to 37 °C using a water jacket and circulator before arriving at the aorta. Approximately 90% of the hearts regained their pink color and spontaneous rhythmic activity. These were therefore studied further. The remaining 10% did not and were discarded. The hearts were perfused for a further 20 min to minimize residual effects of endogenous catecholamine release before their electrophysiology properties were characterized.

### 2.3. Stimulating and Recording Procedures

Paired platinum electrodes (1 mm interpole distance) were used to stimulate the right ventricular epicardium electrically. This took place at 8 Hz, using square wave pulses of 2 ms in duration, with the stimulation voltage set to three times the diastolic threshold immediately after the start of perfusion. The multi-electrode array, which consisted of 64 electrodes (Teflon-coated silver wires; 0.125 mm diameter; Science Products), was arranged in an 8 × 8 configuration (grid dimensions: 1.5 mm × 1.5 mm; electrode diameter: 0.1 mm; inter-electrode distance: 0.21 mm). Signals were acquired at 1.5 kHz, amplified (100 times), and digitized with 4 PXI-6031E cards (National Instrument Inc., Austin, TX, USA). The array was placed against the LV surface with channel 1 near the base of the heart and channel 57 near the apex. The position of the array was determined in a consistent manner using the anatomical landmarks of the left anterior descending artery, the aorta, and the atria. Unipolar electrogram recordings were made from hearts during spontaneous activity, 8 Hz pacing, and S1S2 stimulation. A reference electrode was placed on the opposite ventricle, distant from the recording sites. The electrical signals were stored offline and subsequently analyzed using EMapScope (Version 4.0, MappingLab, Oxford, UK). Isochrones were drawn using the built-in function of the program. From these recordings, conduction parameters were calculated, as described previously by Lammers et al. in detail [26]. The following parameters were obtained: (1) local activation times (LATs), defined as the point of maximal negative slope and displayed in a grid representing the layout of the original recording array [27]. The mean values were taken from five cardiac cycles for each channel, and an overall mean value was taken from all 64 channels. The mean values from all hearts were then averaged; (2) the standard deviation of the mean LATs averaged over five cardiac cycles, across 64 channels was calculated; (3) the median values of histograms of the local maximum phase differences (P_50_); (4) the absolute inhomogeneity in conduction (P_5–95_); (5) the inhomogeneity index given by P_5–95_/P_50_ [26] and (6) P_50_, P_5–95_ and P_5–95_/P_50_ normalized to 1 mm.

### 2.4. Statistical Analysis

All values were expressed as mean ± standard error of the mean (SEM). Numerical data were compared by one-way analysis of variance (ANOVA). *p* < 0.05 was considered statistically significant and was denoted by * in the figures.

## 3. Results

**Figure 1:**
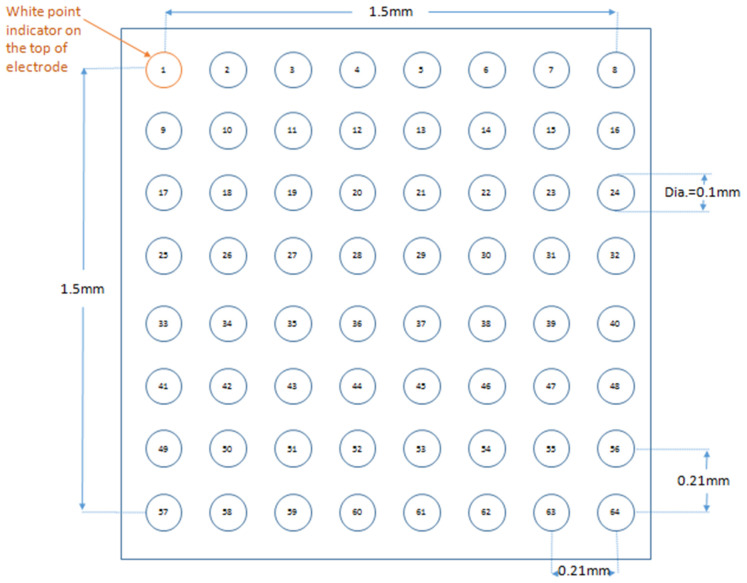
A cartoon of 64-channel multi-electrode array arranged in an 8 × 8 grid. Figure reproduced from MappingLab with permission.

A multi-electrode array was used to investigate the activation patterns of the LV epicardium under different pharmacological conditions. A diagram of the 64-channel multi-electrode array organized in an 8 × 8 configuration is shown in Figure 1. From each channel, a unipolar electrogram was recorded from spontaneously beating hearts during 8 Hz or during S1S2 stimulation applied at the RV epicardium.

**Figure 2:**
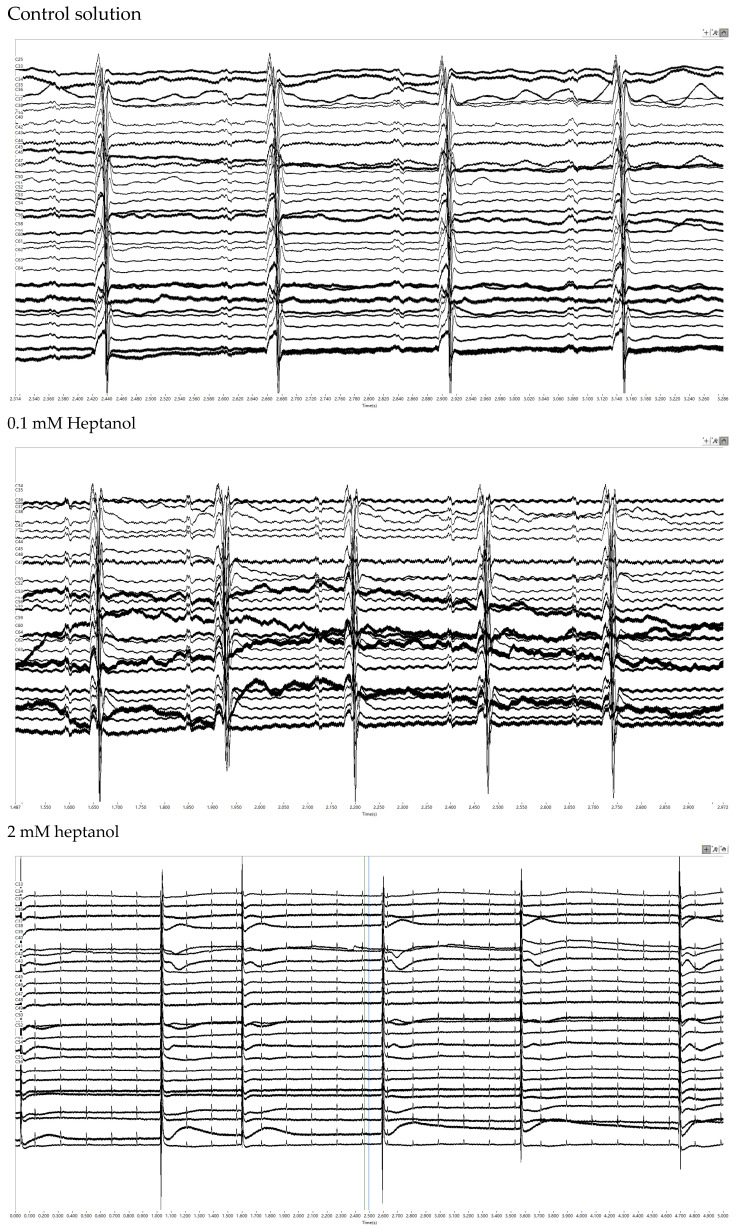
Representative biphasic electrograms obtained from spontaneously beating hearts under control conditions (***top***) and in the presence of 0.1 mM (***middle***) or 2 mM heptanol (***bottom***).

**Figure 3:**
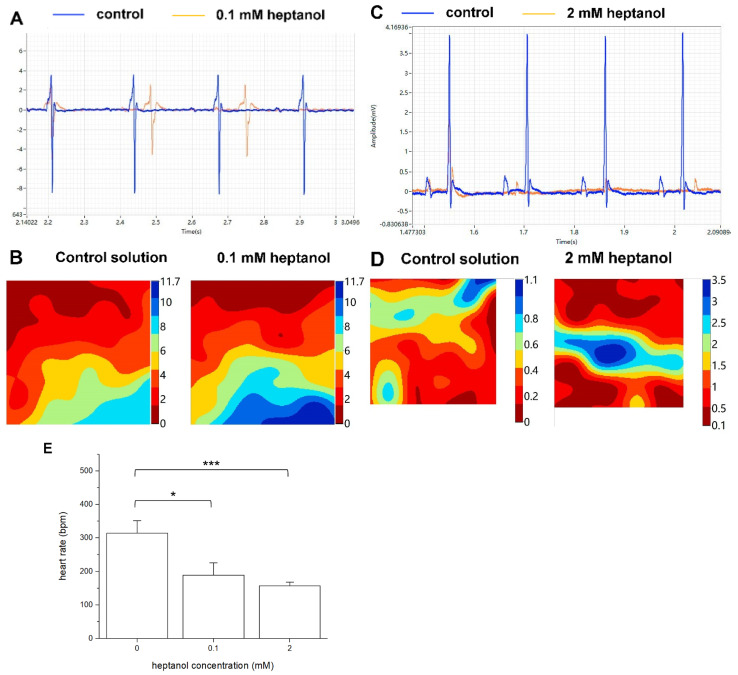
Representative biphasic electrograms from a single channel (**A**) and corresponding activation maps (**B**) obtained from spontaneously beating hearts under control conditions and in the presence of 0.1 mM. Representative biphasic electrograms from a single channel (**C**) and corresponding activation maps (**D**) obtained from spontaneously beating hearts under control conditions and in the presence of 2 mM. There was a dose-dependent reduction in heart rate as heptanol concentration increased (**E**). * *p* < 0.05, *** *p* < 0.001. Data from *n* = 5 hearts. Differences between groups were tested using ANOVA followed by Tukey’s honestly significant difference test.

Representative traces of the electrograms recorded from the spontaneously beating hearts under control conditions showed regular activity (Figure 2, *top panel*). In the presence of 0.1 mM heptanol, ventricular arrhythmias could be detected (Figure 2, *middle panel*). At 2 mM heptanol, regular activity was seen (Figure 2, *bottom panel*). Enlarged traces from a single channel are shown in Figure 3A, whereas the activation maps are shown in Figure 3B for 0.1 mM heptanol. For 2 mM heptanol, the traces are shown in Figure 3C,D, respectively. Heptanol at 0.1 and 2 mM significantly reduced the spontaneous heart rate from 314 ± 25 to 189 ± 24 and 157 ± 7 bpm, respectively (ANOVA, *p* < 0.05 and *p* < 0.001; Figure 3E).

**Figure 4:**
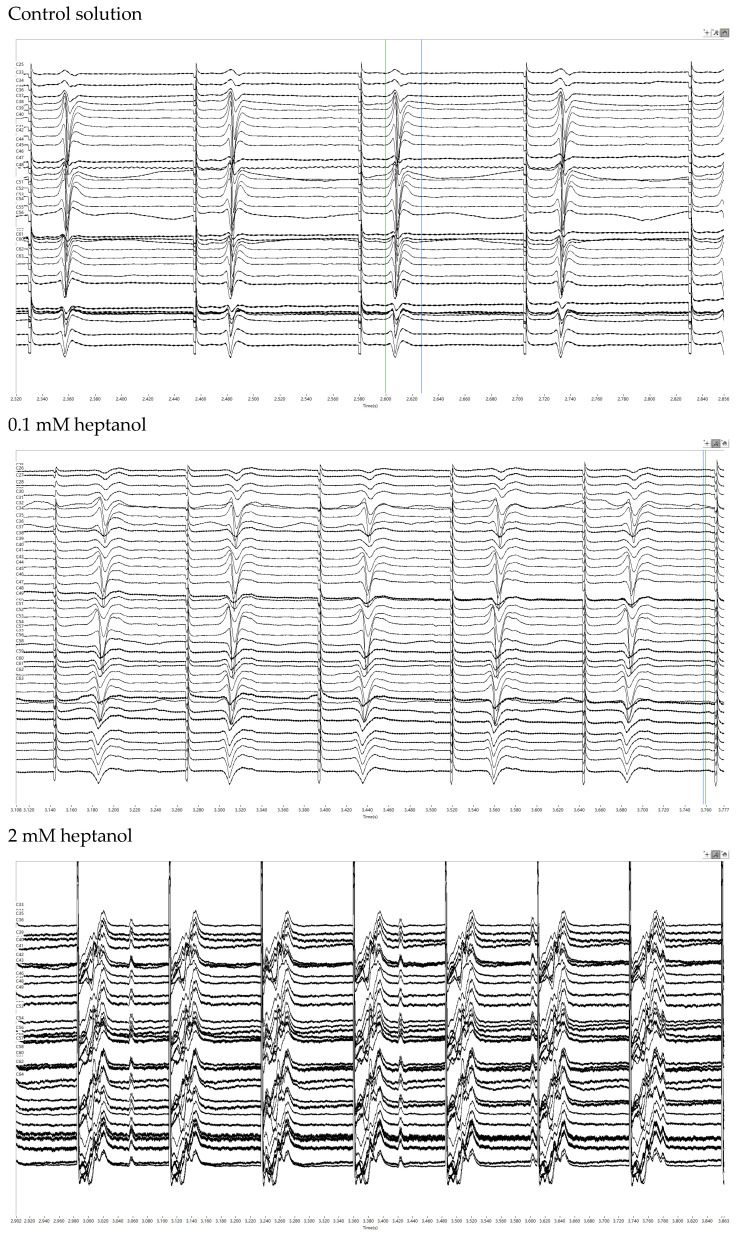
Representative biphasic electrograms obtained during 8 Hz pacing under control conditions (***top***) and in the presence of 0.1 mM (***middle***) or 2 mM heptanol (***bottom***).

**Figure 5:**
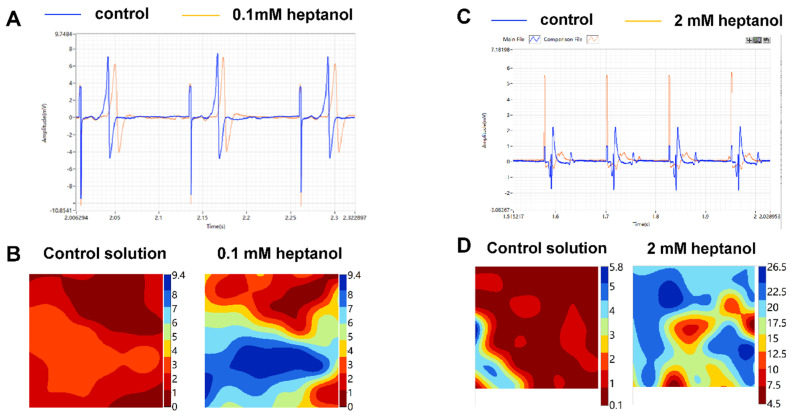
Representative biphasic electrograms (**A**) and corresponding activation maps (**B**) from a single channel obtained during 8 Hz pacing under control conditions and in the presence of 0.1 mM. Representative biphasic electrograms (**C**) and corresponding activation maps (**D**) from a single channel obtained during 8 Hz pacing under control conditions and in the presence of 2 mM.

Subsequent experiments were conducted during 8 Hz pacing to further investigate the electrophysiological properties. The representative traces of the electrograms obtained under control conditions and in the presence of 0.1 mM or 2 mM heptanol are shown in Figure 4. Enlarged traces from a single channel are shown in Figure 5A, whereas the activation maps are shown in Figure 5B for 0.1 mM heptanol. For 2 mM heptanol, the traces are shown in Figure 5C,D, respectively.

**Figure 6:**
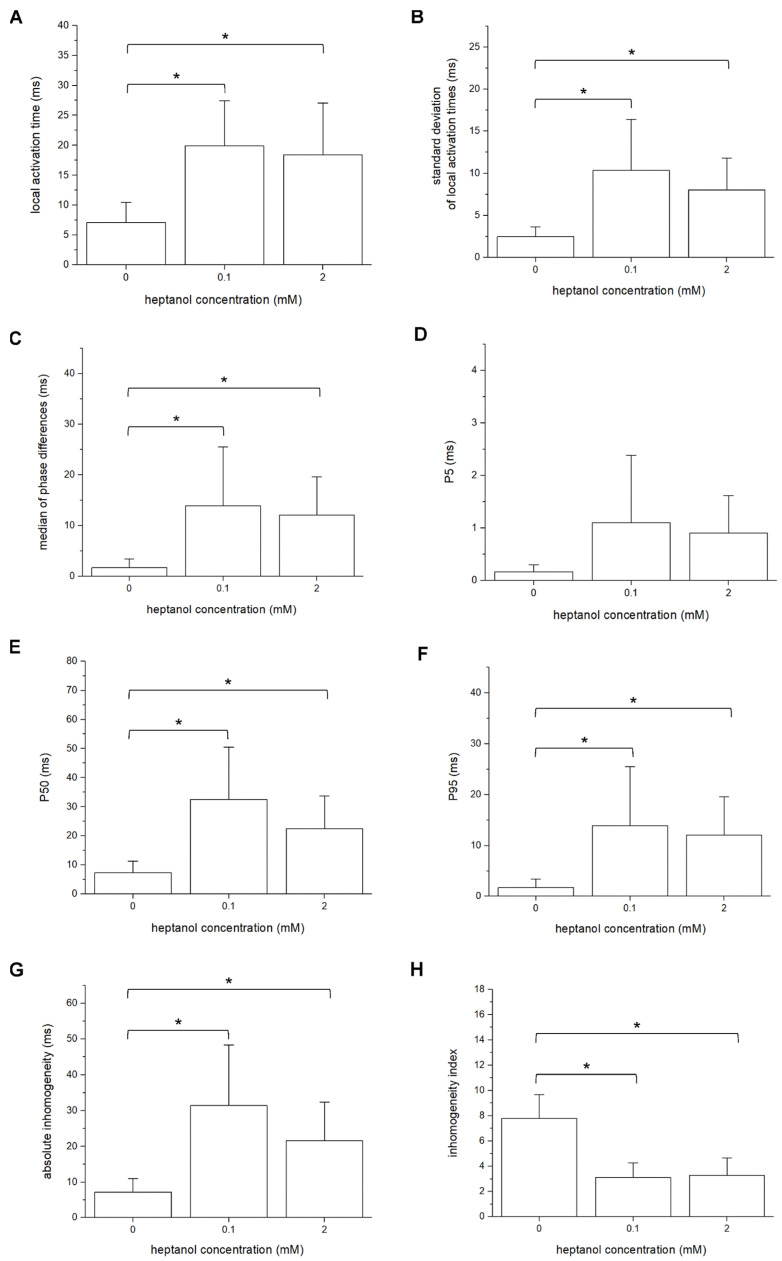
Mean local activation time (LAT) (**A**), standard deviation of mean LATs (**B**), median of phase differences (**C**), P_5_ (**D**), P_50_ (**E**), P_95_ (**F**), absolute inhomogeneity (P_5–95_, (**G**)), and inhomogeneity index (P_5–95_/P_50_, (**H**)) obtained during 8 Hz pacing before and after introduction of 0.1 mM or 2 mM heptanol. Data from *n* = 5 hearts. Differences between groups were tested using ANOVA followed by Tukey’s honestly significant difference test. * indicates *p* < 0.05.

The mean LATs were increased by 0.1 and 2 mM heptanol from 7.1 ± 2.2 ms to 19.9 ± 5.0 ms (ANOVA, *p* < 0.05) and 18.4 ± 5.7 ms (ANOVA, *p* < 0.05), respectively (Figure 6A). The standard deviation of the mean LATs was increased from 2.5 ± 0.8 ms to 10.3 ± 4.0 ms and 8.0 ± 2.5 ms, respectively (ANOVA, *p* < 0.05; Figure 6B), and the median of phase differences was significantly increased from 1.7 ± 1.1 ms to 13.9 ± 7.8 ms and 12.1 ± 5.0 ms by 0.1 and 2 mM heptanol (ANOVA, *p* < 0.05; Figure 6C). P_5_ took a value of 0.2 ± 0.1 ms and was not significantly altered by heptanol at 0.1 or 2 mM (1.1 ± 0.9 ms and 0.9 ± 0.5 ms, respectively, *p* > 0.05; Figure 6D). By contrast, P_50_ was increased from 7.3 ± 2.7 ms to 24.0 ± 12.0 ms by 0.1 mM heptanol and then to 22.5 ± 7.5 ms by 2 mM heptanol (Figure 6E). P_95_ was increased from 1.7 ± 1.1 ms to 13.9 ± 7.8 ms by 0.1 mM heptanol and then to 12.1 ± 5.0 ms by 2 mM heptanol (Figure 6F) (ANOVA, *p* < 0.05 for all). These changes led to increases in the absolute inhomogeneity in conduction (P_5–95_) from 7.1 ± 2.6 ms to 31.4 ± 11.3 ms, 2 mM: 21.6 ± 7.2 ms, respectively (ANOVA, *p* < 0.05; Figure 6G). The absolute inhomogeneity was then divided by the median to determine the inhomogeneity independent of conduction velocity, yielding the inhomogeneity index (P_5–95_/P_50_). This index was significantly reduced from 3.7 ± 1.2 to 3.1 ± 0.8 by 0.1 mM and then to 3.3 ± 0.9 by 2 mM heptanol (ANOVA, *p* < 0.05) (Figure 6H).

**Figure 7:**
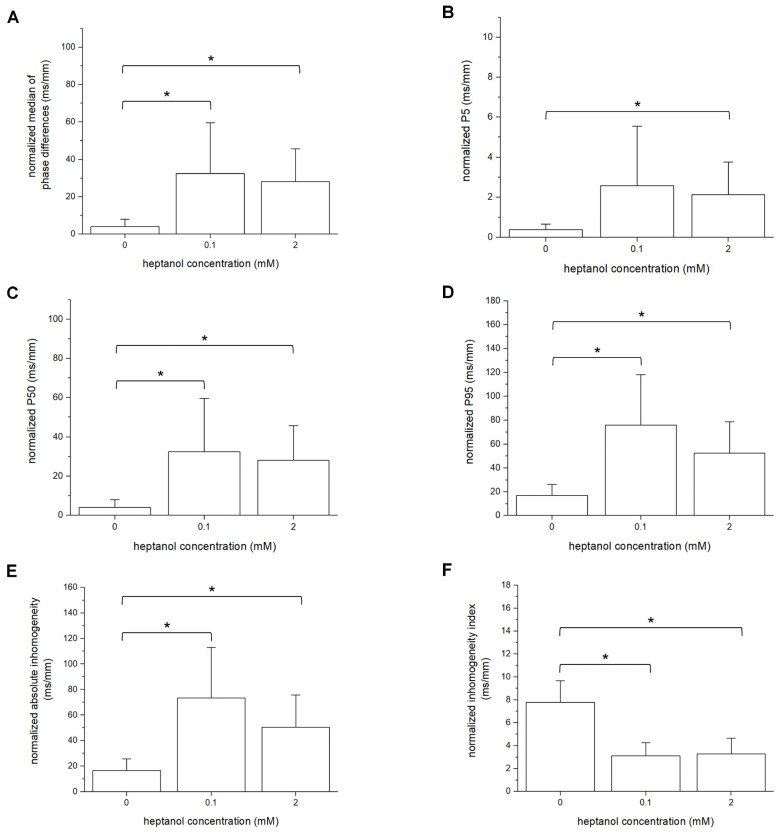
Normalized median of phase differences (**A**), P_5_ (**B**), P_50_ (**C**), P_95_ (**D**), absolute inhomogeneity (P_5–95_, (**E**)), and inhomogeneity index (P_5–95_/P_50_, (**F**)) obtained during 8 Hz pacing before and after introduction of 0.1 mM or 2 mM heptanol. Data from *n* = 5 hearts. Differences between groups were tested using ANOVA followed by Tukey’s honestly significant difference test. * indicates *p* < 0.05.

The different measures of inhomogeneity are also normalized per unit of distance in millimeters. Thus, the normalized median of phase differences under control conditions was 4.0 ± 2.6 ms/mm and was increased to 32.5 ± 18.1 ms/mm and 28.2 ± 11.7 ms/mm by 0.1 mM and 2 mM heptanol (ANOVA, *p* < 0.05 for both cases; Figure 7A). The normalized values of P_5_ were not significantly altered by 0.1 mM heptanol (0.4 ± 0.2 ms/mm vs. 2.6 ± 2.0 ms/mm; ANOVA, *p* > 0.05) but were increased by 2 mM heptanol to 2.1 ± 1.1 ms/mm (ANOVA, *p* < 0.05; Figure 7B). By contrast, normalized P_50_ (Figure 7C) and P_95_ (Figure 7D) were both increased by 0.1 and 2 mM heptanol from 4.0 ± 2.6 ms/mm to 32.5 ± 18.1 ms/mm and 28.2 ± 11.7 ms, and from 17.1 ± 6.2 ms/mm to 75.9 ± 28.1 ms/mm and 52.5 ± 17.4 ms/mm, respectively (ANOVA, *p* < 0.05 for all). These changes led to increases in the normalized absolute inhomogeneity in conduction (P_5–95_) from 16.7 ± 6.0 ms to 73.4 ± 26.4 ms/mm and 50.4 ± 16.8 ms/mm, respectively (ANOVA, *p* < 0.05; Figure 7E). The absolute inhomogeneity was then divided by the median to determine the inhomogeneity independent of conduction velocity, yielding the inhomogeneity index (P_5–95_/P_50_). This index was significantly reduced by 0.1 and 2 mM from 7.8 1.2 mm^−1^ to 3.1 ± 0.8 mm^−1^ and 3.3 ± 0.9 mm^−1^ (ANOVA, *p* < 0.05), respectively (Figure 7F).

## 4. Discussion

In this study, we investigated the contributions of gap junction and/or sodium channel blockade using the pharmacological agent, heptanol. A multi-electrode array was used to determine the activation latencies of 64 myocardial regions simultaneously, which permitted the construction of activation maps and the quantification of both spatial and temporal dispersion of conduction. The main findings are that heptanol at both 0.1 mM and 2 mM concentrations significantly increased local activation latencies (LATs) across myocardial regions, the standard deviations of LATs and absolute inhomogeneity, and decreased the inhomogeneity index.

Multi-electrode arrays can simultaneously record extracellular electrograms from multiple sites. The resulting electrogram data can be used to assess spatial heterogeneities in conduction. The standard deviation of LATs of the different recording channels can be calculated to provide a crude measure of the spread in activation times [28]. To further quantify the degree of inhomogeneity, Lammers and colleagues evaluated phase differences in LATs in the rabbit atria [26]. This enabled the building of histograms of percentile scores of the total population. Theoretically, absolute inhomogeneity in conduction, reflected by P_5–95_, can be a primary abnormality or a secondary one resulting from reduced CVs. To distinguish between these, the absolute inhomogeneity (P_5–95_) can be divided by the median score, P_50_, to calculate the inhomogeneity index (P_5–95_/P_50_). If the inhomogeneity index were unchanged, then the inhomogeneity would be due to lower conduction velocities. If it were increased, then the increased inhomogeneity would be a primary abnormality.

Heptanol is a long-chain alcohol that decreased the fluidity of cholesterol-rich membrane domains, resulting in a reduced open probability of the gap junction channels [29,30] without influencing its unitary conductance [31]. Heptanol inhibited gap junctions reversibly with a K_D_ value of 0.16 mM and a Hill coefficient of 2.3 in guinea pig ventricular cell pairs [32]. In rabbit hearts, a K_D_ value of 0.20 mM and a Hill coefficient of 2.1 were determined [33]. In another study, K_D_ values of 0.54 mM and 1.20 mM were found using the whole-cell and perforated patch recording techniques, respectively, with a Hill coefficient of 3.45 in neonatal rat cardiomyocytes. The IC_50_ value of heptanol for gap junctions is 2.21 mM in HeLa cells expressing the gap junction protein connexin 43 [34]. However, it should be recognized that heptanol at higher concentrations affects the activity of other ion channels located at the plasma membrane. In canine cardiac Purkinje cells, heptanol blocks sodium channels with an IC_50_ of 1.3 mM, with 70% and 100% inhibition at 3 mM and 10 mM, respectively [13]. Heptanol at 0.7 mM was found to reduce the amplitude and dV/dt but not the APD of monophasic action potentials in Langendorff-perfused guinea pig hearts [35]. In the squid axon, heptanol inhibited sodium channels with a K_D_ of 0.93 [36]. Heptanol also inhibited calcium channels at concentrations between 0.5 mM and 6 mM with an IC_50_ value of 0.75 mM and inward rectifier potassium channels at 3 mM, although IC_50_ was not provided [37].

Together, the inhibitory effects on gap junctions and sodium channels explain the conduction slowing and increased dispersion of conduction produced by heptanol [38,39]. In the canine ventricular myocardium, heptanol at 0.5 mM and 1 mM reduced conduction velocity both in the transverse and longitudinal directions, with greater effects in the transverse direction. The authors found that 1.5 mM heptanol produced only a 7% decrease in action potential upstroke velocity (V_max_), suggesting that its effect on the sodium current was negligible in these experimental conditions [40]. In sheep epicardial muscle, heptanol between 1.5 mM and 3 mM produced variable effects on V_max_ but consistently reduced the overall conduction velocity, suggesting an interacting effect between alterations in intercellular coupling and the direction of action potential propagation [41]. The same group found that heptanol applied at concentrations between 1.5 mM and 3 mM reversibly produced a major decrease in conduction velocity and eventually led to conduction block when V_max_ was only reduced by 38% in isolated sheep Purkinje fibers [42]. They further reported that V_max_ at the proximal site was unaltered, whilst V_max_ at the distal site was reduced by 27% following perfusion with 2 mM heptanol. However, conduction block was observed even when V_max_ was relatively normal, suggesting that the effects were mediated through alterations in intercellular resistance. These experimental findings were supported by their computer simulations, which confirmed that increases in intercellular resistance led to reductions in the conduction velocity, even when V_max_ was not significantly altered.

Heptanol can exert varying effects on cardiac arrhythmogenicity depending on the concentrations applied, but also on the cardiac chamber and experimental model used. Thus, heptanol at 0.5 and 1 mM exerted pro- and anti-arrhythmic effects, respectively, in the infarcted canine ventricular myocardium [14]. In isolated rabbit hearts, heptanol produced arrhythmogenic effects at concentrations between 0.1 mM and 0.3 mM [33]. By contrast, in a model of reentrant ventricular tachycardia around a ring of anisotropic myocardium from Langendorff-perfused rabbit hearts, heptanol perfusion at concentrations between 1 mM and 3 mM terminated VT [43]. Interestingly, perfusion with 1 mM heptanol reduced the defibrillation threshold without affecting the repolarization or refractoriness properties [44]. The regional perfusion of 0.5 mM heptanol to swine induced spontaneous ventricular fibrillation and also increased the defibrillation thresholds [45]. These effects are associated with impaired gap junctional conductance and the increased spatial dispersion of conduction. Furthermore, pre-treatment with 1 mM heptanol protects rabbit hearts against ischemia by reducing the infarct size [46]. Heptanol at 0.05 mM, 0.1 mM, 0.5 mM, and 1 mM conferred cardioprotective effects by reducing infarct size following ischemia and prevented the hearts from developing ventricular arrhythmias during reperfusion [47]. However, atrial fibrillation could be induced in the presence of heptanol at low concentrations of 2 μM in isolated perfused canine atria. This effect was attributed to intercellular uncoupling as V_max_ and APD restitution were unaltered [48].

Our previous work found that 2 mM heptanol exerted anti-arrhythmic effects on the atria [49], but pro-arrhythmic effects in the ventricles [15], of isolated mouse hearts. These were attributable to relative changes between conduction and tissue refractoriness, represented by the excitation wavelength. In this study, we found higher values of absolute inhomogeneity given by P_5–95_ induced by heptanol. Moreover, the inhomogeneity index was reduced, suggesting that the inhomogeneity was lower than expected due to conduction slowing. Together, our findings implicate reduced conduction velocities and the increased spatial dispersion of conduction as the substrate for reentrant arrhythmias. Our findings complement previous work in a genetic mouse model of Brugada syndrome, in which temporal and spatial heterogeneities could be assessed by similar multi-electrode array setups [50]. Several factors have been identified as important contributors to spatial heterogeneity in conduction, including the direction of action potential propagation, pacing rate, and premature activation [26].

There are several limitations to the use of extracellular recordings from a multi-electrode array. It cannot distinguish between various mechanisms of conduction block. Nevertheless, some inferences can be made by comparing the phase maps obtained under different conditions. For example, if the inhomogeneities are observed only during premature pacing, then a possible cause is the spatial dispersion of refractoriness [51,52]. Moreover, if the inhomogeneities are present in a single direction only, then they are likely related to tissue anisotropy in axial resistance. By contrast, if they are present in all directions, structural abnormalities may be present. The mechanical movement of the heartbeat can cause the distortion of the electrical waveforms, especially for optical mapping techniques [53]. The motion artefact represents less of a problem for the multi-electrode array technique, because the electrode can move with the heart if the electrode is closely apposed to the heart surface. In our experiments, the electrode pins were made of silver and the outer casing material was made of aluminum. The recordings showed reproducible waveforms over a long period of time. Whilst motion artefact was not a significant problem, the use of flexible electrodes can reduce this problem further. For example, multi-electrode arrays that are composed of flexible materials, such as thin-film polymer, can maintain better conformal contact with the heart motion [54].

Finally, the mechanisms of heptanol at the cellular level, for example, the relative contributions to sodium channel and gap junction inhibition, were not explored in detail, and histological findings were not available.

## 5. Conclusions

Multi-electrode array recordings demonstrated conduction abnormalities in the form of a reduced CV and an increased spatial dispersion of conduction induced by heptanol in Langendorff-perfused mouse hearts.

## Data Availability

Data are available from the corresponding author without restriction.
